# Pneumonia

**Published:** 1894-04

**Authors:** Thomas Turnbull

**Affiliations:** Hartford, Conn.


### PNEUMONIA.

BY THOMAS TURNBULL, JR., M.D., HARTFORD, CONN.

Jurgensen says, outside of phthisis, pneumonia is the most fatal of all diseases.

Pneumonia is an acute, specific, infectious disease caused by a specific germ, the diplococcus pneumoniæ, characterized by inflammation of pulmonary parenchyma and constitutional disturbances of varying intensity.

With Laennec, to whom we owe so much of our knowledge of lung diseases, the idea started "that pneumonia is a local affection, a primary inflammation of the lungs, and the general symptoms in proportion to the extent, profundity and the gravity of the pulmonary lesions." The older writers, ignorant of auscultation, made the pulmonary lesion secondary and subordinate to the general condition, which alone was accessible to them. They held that pneumonia was but the principal localization of a general disease, the peri-pneumonic fever.

Jurgensen thus sums up his opinion of pneumonia in the article on croupous pneumonia in Ziemssen's Cyclopedia, "Croupous pneumonia is a constitutional disease and is not dependent upon a local cause. The inflammation of the lungs is merely the chief symptom, and the morbid phenomena are not due to the local affection. We must admit a specific morbid agent; croupous pneumonia then belongs to the group of the infectious diseases." This was in 1875 and what a step forward it was.

Is pneumonia a general disease? Is pneumonia a disease produced by a specific agent? The second question will be answered first. The experimental physiologists in their experiments in section of the pneumogastrics found that pulmonary lesions follow this operation.

Traube was the first to notice the resemblance of these lesions to those characterizing lobular pneumonia, and showed that it was due to anesthesia of the laryngo-bronchial mucosa allowing liquids from the mouth and even alimentary particles to penetrate into the bronchi and alveoli.

Bretonneau employed irritating vapors, Trasbot and Cornil employed turpentine, Hohenheimer injected purulent septic liquids, even putrefied blood, into the bronchi of dogs on which he had practiced tracheotomy. Wolf injected into the bronchi of hares and guinea-pigs the ordinary bacteria of putrefaction, and yet all these experiments failed to produce a frank pneumonia. A catarrhal pneumonia, or pseudo-lobar broncho-pneumonia was the result. Heidenheim used alternations of heat and cold to produce frank pneumonia but failed, obtaining only tracheitis, bronchitis and nodules of lobular pneumonia; in no case did he observe lobar inflammation or concomitant pleurisy. All the above experiments show that the frank pneumonia cannot have nervous or mechanical origin.

Klebs was the first to describe what he believed to be the specific agent in pneumonia. They were roundish, mobile micrococci. The lesions produced by Klebs, monads however, were those of experimental septicemia in general and not a true pneumonia. Erberth in 1881 described certain round micrococci isolated or in colonies. Koch in 1881 found micrococci in the capillaries of the lungs and kidneys in a case of pneumonia; he was the first to indicate their oval forms. In 1882 Friedlander published his account of the micro-organisms in eight cases of frank pneumonia. This diplococcus, described, cultivated and injected, producing a frank pneumonia, is now usually admitted to be the specific cause of croupous pneumonia.

The second question in regard to pneumonia being a general disease, is not entirely settled yet. Most pathologists look upon it as a local disease, with constitutional symptoms caused by absorption of the pneumotoxine. Probably like diphtheria it starts as a local disease, the constitutional symptoms following the poison absorption, or it may be reasoned that the poison enters by the lungs; the constitutional symptoms spring up followed by the pulmonary symptoms. In most cases it will usually be found that there were prodromal lung symptoms.

Pneumonia is both infectious and epidemic. Epidemics are described as occurring in 1348, 1585, 1621, 1708, 1768, and several since then. In the Kentucky State Prison Hospital in 1875, seventy-five of the prisoners had pneumonia with a mortality of eight per cent. In 1876 another epidemic occurred in the same prison when twenty-five out of ninety-eight succumbed. City and town epidemics are quite common. We are all familiar with the house epidemics when entire families are wiped out.

Among the conditions favoring the development of pneumonia may be mentioned the seasons, climate, winds, hygrometric states, putrid miasms and sewer-gas, chilling of the body, traumatism, overwork and overcrowding. Besides these there are the individual conditions, sex, age, habits, etc.

The pathological anatomy will not be taken up as you are all familiar with the three stages of the inflamed lung.

*Course.*—Pneumonia is a typical disease, taking all cases into consideration. The subjective and objective symptoms, due to the local affections of the lung, usually take chief place among the clinical appearances. Like the other infectious diseases it runs a certain course, extending over a certain time and ending usually by crisis. The length of the attack varies according to the severity, individual circumstances and complications, lasting usually from three to seven days.

*Symptoms.*—The slight prodromal symptoms I wish to call attention to, as we are overlooking them since we acknowledged the specific cause. The chill usually is sharp and makes a marked impression on the patient, so much so that the slight prodromes are forgotten and the chill is given as the initial symptom.

In the influenzal pneumonia the chill is often lacking, slight chilly sensations taking its place. The pain in the side is not marked and is taken for the thoracic pains of influenza, the sputa is simply mucous and catarrhal, and we think it a case of bronchial influenza, when all the symptoms are suddenly intensified and we have the later stages of a frank pneumonia. In many cases during the past winter this is what we have had, with much more fatal results than in true pneumonia.

Temperature, pain, respiratory, nervous and cerebral symptoms you are all familiar with, and they need only be mentioned in passing.

The complications of pneumonia are few, being only pleurisy, peri and endocarditis, peripheral neuritis, phlegmasia alba dolens, which is quite common. Serious gastric and enteric complications are rare. Parotitis occasionally occurs. Bright's disease does not often follow, but pneumonia is a frequent complication of Bright's. Peritonitis is exceedingly rare. Relapse is uncommon. "Wagner in eleven hundred cases met only three relapses, and these were doubtful." Recurrence is more common in pneumonia than in any other acute disease, many persons having four or five attacks.

The termination of pneumonia in favorable cases is resolution, which usually sets in shortly after the crisis. The other terminations, which are unusual or rare, are abscess, gangrene and fibroid induration.

*Diagnosis.*—Pneumonia is usually one of the easiest of all diseases to diagnose. The greatest trouble is in old people and drunkards. The delirium of drunkards with pneumonia is apt to be mistaken for delirium tremens and the pneumonia overlooked. All these cases should be examined for pneumonia. In old persons and during an influenza epidemic many of the symptoms are masked and a diagnosis is difficult. Pleurisy with effusion is sometimes, in children, taken for pneumonia, and often the first few breaths taken by a person rising from a dorsal decubitus give a crepitant râle which is often very misleading, and has been diagnosed as pneumonia. By making the patient take a few long, deep breaths this passes quickly away, as it is only due to hypostatic congestion. The crepitant râle is no longer considered an infallible sign, as it is found in other conditions. Pneumonia is often secondary to chronic troubles, and is often overlooked, being taken for an exacerbation.

Prognosis depends to a certain extent upon the age, rising from 3.7 per cent. under twenty years to 22 per cent. in the third decade, 30.8 per cent. in the fourth, 47 per cent. in the fifth, 51 per cent. in the sixth and 65 per cent. in the seventh decade. In children the outlook is good. Endocarditis is a grave complication ; meningitis is usually fatal. Early signs of heart failure, cyanosis and difficulty in breathing, with mucous râles, are symptoms of gravity. Death is, in the majority of cases, due to heart failure, whether induced by fever, specific action of poison, or paralysis due to overdistention of right ventricle.

*Treatment.*—Pneumonia, being a self-limited, specific disease, is at present uninfluenced in its course by any drug. The latest specific treatment is that of G. and F. Klemperer, who found that rabbits, vaccinated with varying strength of bouillon cultures of the pneumococci, became immune against stronger injections. They then isolated an albumose pneumotoxine, which being injected into rabbits and man produced febrile symptoms similar to pneumonia; in the previously inoculated rabbits the pneumotoxine had no effect. "In addition they found that the effect of injections of cultures of pneumococci was promptly antagonized by the injection of serum of men rendered immune by a recent attack of pneumonia. The time that had elapsed in the men from whom the serum was obtained, after the crisis, varied from one day to three months, and in all these cases the same results followed the injection of the serum." "The explanation of these facts was that the blood of immune persons contains a substance which they called antipneumotoxine, antidotal to the pneumotoxine, developing during the course of the disease, owing to some action of the pneumotoxine upon the serum, until it is present in sufficient quantity to neutralize the pneumotoxine, and thence persisting during the period of immunity." The Kemplerers report six cases with the crisis appearing in from six to twelve hours after injection, with slowing of pulse and respiration. The quantity of serum was from 4—6 c. c. Neissner reports three cases. Redner reports twenty cases with uniform recovery. Jensen has reported favorable cases. Hughes and Carter, of Philadelphia, report a case successfully treated by the serum injections. Here in over thirty reputed cases we have recovery in all, with only five cases in which the crisis appeared at such a time as rendered the effect of the injections doubtful.

Now specific treatment has changed from the intense anti-phlogistic treatment of the first part of the century and the succeeding do-nothing treatment of the past few years into a rational conservative position of making way early for the symptoms which are to follow. Outside of the specific infection the treatment of pneumonia is the treatment of an overworked, overdistended right heart. Our main object is to keep this strong and relieve the venous vessels by dilating the arteries and equalizing the blood-current. This is best done in the first stage by aconite. Wood says "on the heart aconite acts as a quieter of its movements and force, and so lowers blood-pressure and pulse-rate by a direct action in the heart. There is no evidence of its possessing any direct influence on the vaso-motor system." It lowers temperature by increasing heat radiation. We thus see that aconite quiets the heart and prepares it for the struggle which is to come. After the first stage aconite is considered dangerous by most authors, and as its action on the vaso-motors is slight, it had better be dropped and some of the vaso-dilators substituted. Here nitroglycerine comes into play. Where we have cyanosis, labored breathing, mucous râles, rapid weak pulse and all the signs of a weakened right heart, with a decrease of the accentuation of the pulmonary second sound, nitroglycerine in large doses will so distribute and equalize the blood-current, emptying the veins and filling the arteries, that it sometimes appears magical the way the bad symptoms disappear and the relief to the patient is very marked. The action of nitroglycerine is fugacious, the effects of a dose lasting only from thirty to forty-five minutes, so it must be repeated frequently. It can be used with safety in doses of one one-hundredth of a grain every hour. The pulse is not a guide for the administration of nitroglycerine; as soon as the pulmonary valve sound begins to weaken it should be pushed.

Strychnine is very useful and though opposed in action to nitroglycerine, its action directly on the heart muscle is beneficial and will overcome the slight depressant action of the nitroglycerine. The fever of pneumonia seldom requires treatment; if excessively high, careful administration of phenacetine or cold sponging will reduce it. Feeding is an important point. There is a demand of the system for liquids and water should be freely given. The system is clogged, there is no demand for food. The stomach cannot take care of much, and as the course of the disease is short there is no need of filling our patient full of food he cannot digest. Physiologically the stomach takes from three to four hours to empty itself, so to keep from over-loading the stomach food should not be given at less than three hour intervals, and then only in small amounts. Andrew H. Smith says, "Giving an excess of food therefore entails a double embarrassment. There is the burden arising from undigested food in the stomach, giving rise to flatulent distention, and thus rendering respiration more difficult, and there is also the risk of loading the blood with more nutritive material than the imperfect respiration can act upon in the process of sanguification. In regard to this latter point, I think it more important than it generally appears to have been regarded. We are too apt to overlook the fact that before the food so absorbed can really contribute to the sustenance of the body or add to the strength of the patient, it must undergo a process of assimilation, a process in which respiration plays an important part." Really the most important thing is just to give enough food to sustain the forces without burdening the system to throw off excess. Alcohol is useful in that it acts as a food, is a vaso-motor dilator, any excess is oxidized and easily thrown off and does not clog the system, and above all it is a direct cardiac stimulant. Judiciously given alcohol is one of our most valuable remedies. For the pain in the side nothing is better than a hypodermic of morphia and atropia, which stops pain, relieves embarrassed respiration and stops cough. For cough alone small amounts of dover's powders are beneficial. For the insomnia chloral-hydrate may be given but needs to be carefully watched. Paraldehyde in one-half dram doses, in suspension, acts very happily in many cases. Convalescence is usually rapid and recovery complete.

---

TREATMENT OF GONORRHEA.—W. S. James recommends the following injection, which, according to him, has given excellent results in a case of chronic gonorrhea, where sulphate of zinc, nitrate of silver, and bichloride of mercury had proved inefficient. He says that he has obtained equally favorable results from this composition in the acute form of this disease.

℞—Boracic acid, ʒ iss.; tincture of iodine, ʒ ij.; glycerine, ʒ j.; distilled water, q. s. ad. ℥ iv. To be used as an injection, morning and night.—*International Journal of Surgery.*

